# Communications from Practice

**Published:** 1883-07

**Authors:** 

**Affiliations:** Laboe, Holstein


### COMMUNICATIONS FROM PRACTICE.

### DR. HANNES, LABOE, HOLSTEIN.

In September, 1881, I treated a young lady of seventeen for scarlet fever. At first, in addition to the strongly developed eruption, there was only moderate fever and inconsiderable angina. Later there occurred a dry, irritating cough. It began usually in the afternoon and continued into the evening, and even into the night, while during the forenoon she was tolerably free. By degrees there set in great restlessness, increased fever, sleeplessness, *annoying dryness of the mouth, shortness of breath, and violent, pressing pain in the breast* aggravated by deep respiration. There also followed *a remarkable sleeplessness with difficult speech, as if the tongue was also moved with difficulty.* The sensorium appeared to be unaffected. The remedies used at first (Drosera and Hyoscyamus) were ineffectual. I then gave Nux moschata$^{30}$, in solution. After the first dose (in the evening) she soon slept, and awoke next morning free from all complaint. The fever, cough, etc., did not return again and she recovered rapidly.

A countryman consulted me about his wife March 22d, 1882. According to his statements, she had recently aborted in the fifth month, the afterbirth had not passed, and she was suffering from labor-like pains. I prescribed Pulsatilla$^{30}$. The afterbirth passed in the next day or two, and the pains ceased. However, heat, thirst, cough, and inclination to vomit set in. I again prescribed, on the husband's report, Bryonia$^{30}$. Some days after I was called in on account of the patient becoming worse. I found the formerly robust and blooming woman in the following condition: Pale, sunken face; rapid, short, superficial breathing; short cough, with rattling of mucus, yet she was unable to expectorate anything; very rapid, small, soft pulse; heat, thirst, *dryness in the mouth*, *burning pain in the breast* above the pit of the stomach; great weakness; there were also frequent *attacks of oppression of the chest* with *rapid respiration* and a sensation as if the mucus obstructed the lungs; *violent palpitation, faint-like weakness, and inability to speak.* Except during these paroxysms she is able to speak in a weak voice. Physical examination of the breast and abdomen revealed nothing important, particularly there was nowhere painfulness on pressure, only auscultation discovered extensive coarse *rales* in the region of the right shoulder blade.

I prescribed Nux moschata$^{30}$ in water, hourly, and made a doubtful prognosis.

Report on the following day considerable improvement after a few hours; could expectorate easily; respiration free; paroxysm has not returned.

Three days after is very well, only yesterday she had a short paroxysm. Nux mos.$^{30}$ She soon reported that she was again restored.

Nux mos. is also frequently indicated in children as well as in women, particularly in colic-like attacks with transient *paleness of the face, weakness, sleepiness, faintness,* and the like. It is certainly more frequently indicated than the routine administration of Santonine lozenges or even Cina$^{30}$ in the supposed worm troubles.

I was consulted in May, 1881, by a countryman of sixty-three. He has suffered for several years from rheumatic pains in the right leg from the hip to the foot. Particularly in the evening *in bed*, with trembling and "flying" of the leg, *restlessness and tossing about.* After he becomes warm he sleeps. Morning, on rising, he is stiff; *on moderate motion the pains are more tolerable than sitting still,* yet he cannot walk long. He formerly had rheumatic pains in the left arm and left breast, which also was worse in bed, with restlessness and tossing about.

I prescribed Ferrum oxydat. 6 dec. a dose a day. Four weeks afterwards: "Considerable improvement, in the first eight days little change, then marked improvement, then again a slight aggravation, and again increasing amendment. Pains are now inconsiderable, can work." Again Ferrum oxy. 6 dec. The pains then disappeared entirely and have not yet returned, as he recently informed me.

On the evening of November 2d, 1881, I was called to see the young wife of a countryman. She had had for some days light drawing pains in the right leg from the hip downward. On that day at noon the pains became worse suddenly, and are increasing. *Violent, drawing, tearing pains* from the loins along the anterior surface of the right thigh downward, *aggravated by every movement.* "Cannot move the leg." The ischio-sacral region on the right side is painful on heavy pressure. Accompanying are *heat, restlessness, irritability, thirst, red, hot cheeks, one particularly.* Cham.$^{30}$ in solution. She slept soon after the first dose, and on the following day I found her walking about her room free from pain.

On the 16th of April, 1882, a fisherman's wife consulted me. She had been suffering for four weeks from gastrodynia. It is worse from 3 or 4 P. M. till late in the evening, and *after every meal,* after a few bites, the pains become more violent; she *must loosen the clothing about the* pit of the stomach and press firmly thereon with her hand. Eructations ameliorate but little; feels best when she is warm in bed; less pain at night. The pains go from the back through the left hypochondrium to the pit of the stomach. *Loss of appetite, tongue coated whitish,* constipation, *urine brown;* dirty, yellow, sunken face; whites of the eyes yellow; pit of the stomach painful on pressure. Lycop.$^{30}$ every three days.

April 25th, she sent following report: Violent pains day and night in the bowels and back; complete loss of appetite; nausea; stool bright yellow; urine brown, as if mixed with blood—Chelidonium$^{30}$ every three days. On the 3d of May she came herself. Considerable improvement in every regard; the pains had abated immediately; urine almost clear; stool natural, yet yellow; whites of the eyes clear; tongue clean; pain in the pit of stomach only after eating, but not amounting to much; pains in back have disappeared; sleep, appetite, etc., good. In the upper part of right hypochondrium still sensitive to pressure, on that place the pains are the worst. Sacc. lac. The remainder of her complaints soon disappeared and she still feels entirely well.—*Allegemeine Homœopathische Zeitung. Translated by A. McNeal, Jeffersonville, Ind.*

